# Aspartate aminotransferase of Rhizobium leguminosarum has extended substrate specificity and metabolizes aspartate to enable N2 fixation in pea nodules

**DOI:** 10.1099/mic.0.001471

**Published:** 2024-07-29

**Authors:** Raphael Ledermann, Alexandre Bourdès, Marion Schuller, Beatriz Jorrin, Ivan Ahel, Philip Simon Poole

**Affiliations:** 1 Department of Biology, University of Oxford, OX1 3RB, Oxford, UK; 2 John Innes Centre, NR4 7UH, Norwich, UK; 3 School of Animal and Microbial Sciences, University of Reading, RG6 6AJ, Reading, UK; 4 Sir William Dunn School of Pathology, University of Oxford, OX1 3RE, Oxford, UK

**Keywords:** AatA, amino acid metabolism, nitrogen fixation, *Rhizobium*, symbiosis

## Abstract

*Rhizobium leguminosarum* aspartate aminotransferase (AatA) mutants show drastically reduced symbiotic nitrogen fixation in legume nodules. Whilst AatA reversibly transaminates the two major amino-donor compounds aspartate and glutamate, the reason for the lack of N_2_ fixation in the mutant has remained unclear. During our investigations into the role of AatA, we found that it catalyses an additional transamination reaction between aspartate and pyruvate, forming alanine. This secondary reaction runs at around 60 % of the canonical aspartate transaminase reaction rate and connects alanine biosynthesis to glutamate via aspartate. This may explain the lack of any glutamate–pyruvate transaminase activity in *R. leguminosarum*, which is common in eukaryotic and many prokaryotic genomes. However, the aspartate-to-pyruvate transaminase reaction is not needed for N_2_ fixation in legume nodules. Consequently, we show that aspartate degradation is required for N_2_ fixation, rather than biosynthetic transamination to form an amino acid. Hence, the enzyme aspartase, which catalyses the breakdown of aspartate to fumarate and ammonia, suppressed an AatA mutant and restored N_2_ fixation in pea nodules.

## Introduction

**Fig. 1.**
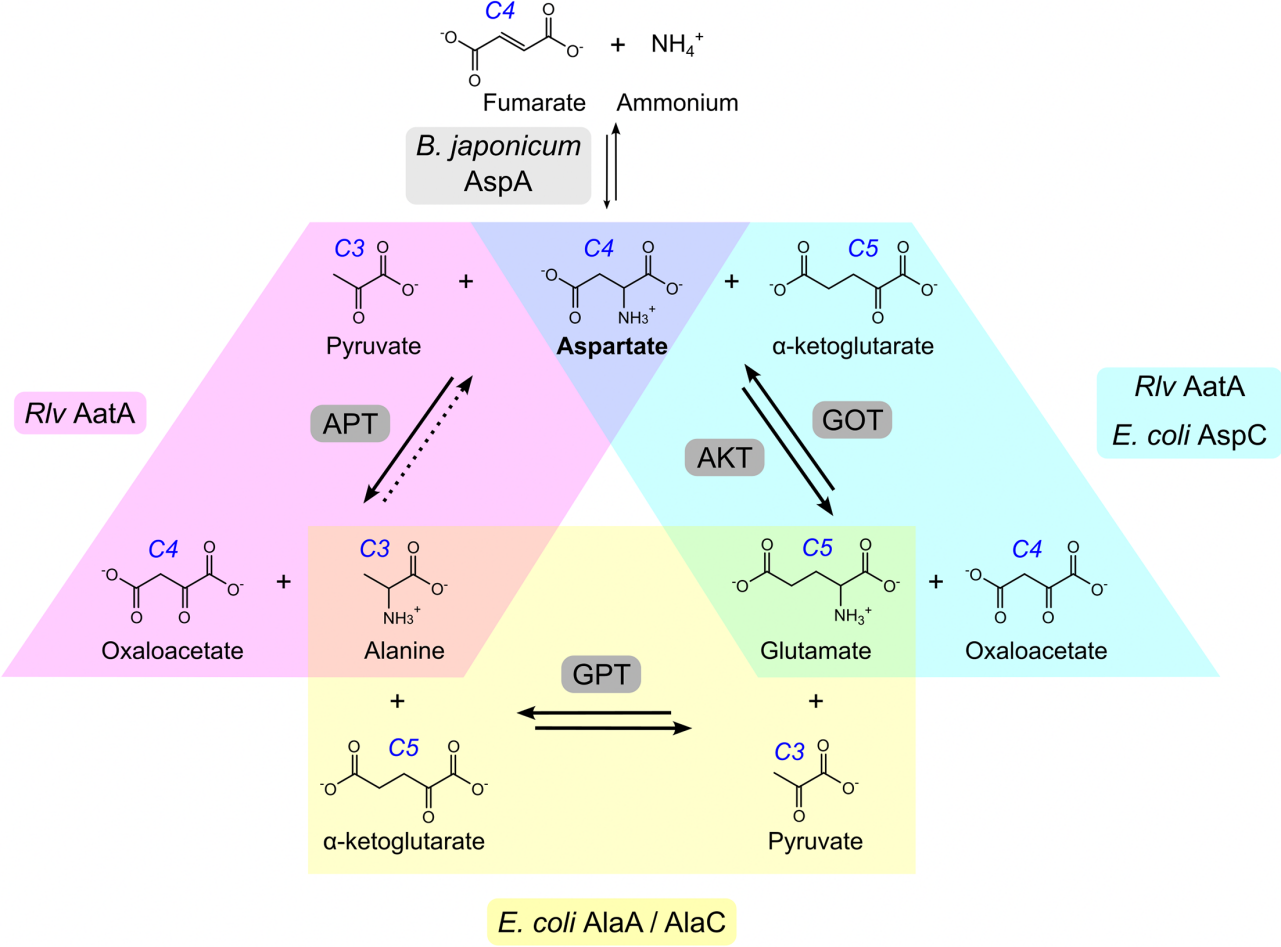
Overview of reactions referred to in the manuscript. The reactions catalysed by both *Rhizobium leguminosarum* bv. *viciae* (*Rlv*) AatA and *Escherichia coli* AspC are shown in cyan (AKT and the reverse GOT). The additional reactivity of AatA compared to AspC described in this work is shown in magenta [aspartate–pyruvate transamination (APT)]. We were unable to detect any reverse APT activity of AatA (indicated by a dashed arrow). The reversible reaction linking glutamate and alanine directly [glutamate–pyruvate transamination (GPT)], found in eukaryotes and *E. coli* (catalysed by AlaA and AlaC) but which we found absent in *Rlv*, is shown in yellow. The reaction catalysed by aspartases (aspartate ammonia lyases), e.g. *Bradyrhizobium japonicum* AspA, is shown above the central aspartate.

Ammonium (NH_4_^+^) is the simplest inorganic nitrogen source for bacteria and can be assimilated directly into amino acids via glutamine synthase (GS) and glutamine oxoglutarate transaminase (GOGAT). The resulting product, glutamate, acts as a universal amino group donor for various transamination reactions involved in the biosynthesis of other amino acids. A second important amino group donor is aspartate [1], which is involved in the biosynthesis of asparagine, threonine, arginine, methionine, lysine, purines, pyrimidines and other metabolites. Aspartate can be synthesized by transamination of the TCA cycle intermediate oxaloacetate from glutamate, forming α-ketoglutarate in the process. The responsible enzyme, aspartate aminotransferase (AatA), catalyses the forward and reverse reactions, forming both glutamate and aspartate [aspartate–α-ketoglutarate transamination (AKT) and glutamate–oxaloacetate transamination (GOT) activity, respectively] (Fig. 1), thus directly linking the pools and fluxes between the two most important amino group donors [2].

Alanine can likewise be produced by the glutamic–pyruvic transaminases [glutamate–pyruvate transamination (GPT)] (Fig. 1). GPT activity is usually high in eukaryotic cells, typically reaching rates of several hundred nmol min^−1^ mg protein^−1^ in both animal [3,4] and plant [5,6] tissues. GPT enzymes AlaA and AlaC are also found in *Escherichia coli* participating in alanine biosynthesis [7]. However, compared to eukaryotic tissues, GPT activity measured in *E. coli* is at least one order of magnitude lower [8]. Whilst many Gamma- and Betaproteobacteria contain genes encoding GPT enzymes, they are largely absent in Alphaproteobacteria, including *Rhizobium leguminosarum* bv. *viciae* (*Rlv*) [9,10]. However, *Rlv* possesses an alanine dehydrogenase (AldA), which directly aminates pyruvate using ammonium, yet deletion of *aldA* does not cause alanine auxotrophy, indicating the presence of alternative alanine biosynthetic pathways, likely involving uncharacterized transaminases [11]. Alanine becomes one of the most abundant amino acids in nitrogen-fixing bacteroids [12], the symbiotic state of rhizobia within root nodules of host legume plants [13,14]. In addition to the nitrogenase product ammonium, alanine is also secreted from bacteroids in increasing amounts as the oxygen tension is lowered [15,16]. Therefore, alanine biosynthesis has attracted considerable attention in the past [17]. However, mutants in AldA were found to exhibit a wild-type-like phenotype in symbiosis with pea plants [11].

In addition to alanine and ammonium, aspartate is also released by bacteroids to host plants [15,18,21]. As bacteroids show reduced flux of carbon towards α-ketoglutarate [15], the assimilation of ammonium into glutamate is restricted. Nonetheless, *aatA* has been found to be essential for proper bacteroid functioning [18,22]. As the reaction catalysed by AatA is reversible, it can form either aspartate or glutamate. Moreover, amino acids are not necessarily formed endogenously by bacteroids but can also be provided by the host plant [23,25]. Thus, the role of AatA in the symbiosis between *R. leguminosarum* and pea plants has remained enigmatic.

Here, we show that AatA of *R. leguminosarum* exhibits an additional activity, forming alanine by transaminating pyruvate using aspartate as a donor, thereby allowing for the indirect transamination of pyruvate from glutamate without the need for GPT enzymes. Moreover, we show that the role of AatA in symbiosis is aspartate degradation as a Δ*aatA-*mutant phenotype can be suppressed by the *Bradyrhizobium japonicum* aspartase AspA, which breaks down aspartate to fumarate and ammonia.

## Methods

### Bacterial strains and cultivation

*E. coli* strains were grown in lysogeny broth (LB) at 37 °C [26] with appropriate antibiotics at the following final concentrations (in µg/ml): ampicillin 100, kanamycin 50, spectinomycin 50, streptomycin 50 and gentamicin 10. Strain ST18 was supplemented with 50 µg ml^−1^ 5-aminolevulinic acid.

*R. leguminosarum* bv. *viciae* was either grown in complex tryptone - yeast extract (TY) medium [27] or universal minimal salts (UMS) medium [28] at 28 °C with appropriate antibiotics added at the following final concentrations (in µg/ml): streptomycin 500, neomycin 80 (for initial selection) or 40 (for routine growth) and gentamicin 20. All strains and plasmids used in this study are listed in Table 1.

**Table 1.** Bacterial strains and plasmids

Strain or plasmid	Relevant genotype or phenotype	Source
*E. coli*		
DH5α	F^-^ *supE44* Δ*lacU169* (φ80 *lacZ*ΔM15) *hsdR17 recA1 gyrA96 thi-1 relA2*	BRL, Gaithersburg
TOP10	Sm^r^ Sp^r^ F^-^ *mcrA* Δ(*mrr-hsdRMS-mcrBC*) φ80 *lacZ*ΔM15 Δl*acX74 recA1 araD139* Δ(*ara-leu*)*7697 galU galK* λ^-^*rpsL endA1 nupG*	Invitrogen
ST18	Sm^r^ *pro thi hsdR*^+^ chromosome::RP4-2 Tc::Mu-Kan::Tn*7*/λpir Δ*hemA*	[58]
BL21 Star (DE3)	F^-^ *ompT hsdS*_B_ (r_B_^-^m_B_^-^) *gal dcm rne131* (DE3)	Invitrogen
*R. leguminosarum* bv. *viciae*		
A34	Sm^r^ wild-type	[59]
RU1640	Sm^r^ Nm^r^ *aatA*::Tn*GeneJumper*	[18]
OPS2038	Sm^r^ Δ*aatA*	This work
OPS2127	Sm^r^ Gm^r^ Δ*aatA* Tn*7*::P_J23104_-e*YFP*	This work
OPS2128	Sm^r^ Gm^r^ Δ*aatA* Tn*7*::P*_aatA_-aatA*	This work
OPS2129	Sm^r^ Gm^r^ Δ*aatA* Tn*7*::P*_aatA_-_Ec_aspC*	This work
OPS2134	Sm^r^ Gm^r^ Δ*aatA* Tn*7*::P*_aatA_-_Bj_aspA*	This work
Plasmids		
pJQ200SK	Gm^r^ *sacB oriT oriV*(P15A) *lacZα*	[60]
pUC18T-mini-Tn7T-Gm	Ap^r^ Gm^r^ *oriT oriV*(ColE1) Tn*7* delivery vector	[61]
pTNS3	Ap^r^ *oriT oriV*(R6K) *tnsABCD* Tn*7* helper plasmid	[62]
pOGG024	Gm^r^ pL1V-Lv1-gent-pBBR1-ELT3 medium copy, broad-host range Level 1 cloning vector	[63]
pOGG072	Sp^r^ pL0V-SC destination vector	[64]
pOGG120	Sp^r^ pL0M-P P_J23104_	[65]
pOGG143	Sp^r^ pL0M-U Rstd	[65]
pOGG157	Sp^r^ pL0M-T DT16	[63]
pET101/D-TOPO	Ap^r^ *lacI* V5-tag 6xHis *oriV*(ColE1) *rop*	Invitrogen
pOPS1349	Gm^r^ (pJQ200SK) *aatA* up- and downstream regions	This work
pOGG130	Sp^r^ pL0M-SC *eYFP*	This work
pOPS0696	Gm^r^ pL1V-J23104-Rstd-*eYFP*-DT16	This work
pOPS1016	Ap^r^ Gm^r^ (pUC18T-mini-Tn7T-Gm) P_J23104_-e*YFP*	This work
pOPS1377	Ap^r^ Gm^r^ (pUC18T-mini-Tn7T-Gm) P*_aatA_-aatA*	This work
pOPS1378	Ap^r^ Gm^r^ (pUC18T-mini-Tn7T-Gm) P*_aatA_-_Ec_aspC*	This work
pOPS1383	Ap^r^ Gm^r^ (pUC18T-mini-Tn7T-Gm) P*_aatA_*-_Bj_*aspA*	This work
pRU1152	Ap^r^ (pET101/D-TOPO) *aatA*	This work
pRU1471	Ap^r^ (pET101/D-TOPO) *_Ec_aspC*	This work

Ap, ampicillin; Gm, gentamicin; Km, kanamycin; Nm, neomycin; rdenotes resistanceSm, streptomycin; Sp, spectinomycin

### Strain construction

To generate a markerless in-frame deletion mutant of *aatA* in *Rlv* A34, 750 bp of the *aatA* up- and downstream region was PCR amplified, including the first four and last five *aatA* codons (including the stop codon) using primer pairs oxp3538/oxp3539 and oxp3540/oxp3541, respectively (see Table S1, available in the online version of this article, for any primer sequences used in this study). The resulting fragments were cloned into *Bam*HI- and *Spe*I-digested pJQ200SK using HiFi assembly (NEB). The resulting plasmid pOPS1349 was mobilized into *Rlv* A34 via biparental mating. Single crossover exconjugants were selected on TY supplemented with gentamicin and propagated without antibiotics, and double crossover candidates were selected on TY supplemented with 5 % (w/v) sucrose. Correct deletion of *aatA* in the final strain OPS2038 was verified using primer pair oxp3638/oxp3639, which amplifies the whole deletion region and primer pair oxp3640/oxp3641 as a negative control, which amplifies an internal fragment of the deleted region. To generate complementation constructs, pUC18T-mini-Tn7T-Gm was digested with *Bam*HI and *Hin*dIII. The endogenous *aatA* promoter of *Rlv* A34 was amplified using primer pair oxp3534/oxp3535. Candidate genes were amplified from gDNA as appropriate: *aatA* from *Rlv* A34 using primer pair oxp3536/oxp3537, *aspC* from *E. coli* DH5α using primer pair oxp3528/oxp3529 and *aspA* from *B. japonicum* 110*spc*4 [29,30] using primer pair oxp3753/oxp3754. The P*_aatA_* fragment was assembled with each amplified gene into the prepared backbone by HiFi assembly, generating plasmids pOPS1377, pOPS1378 and pOPS1383, respectively. To generate a negative control Tn*7* insertion plasmid, first, the *eYFP* (synthetic) ORF was assembled into pOGG072 using *Bpi*I in a Golden Gate reaction, generating pOGG130. In a *Bsa*I Golden Gate reaction, pOPS0696 was then assembled using pOGG120, pOGG143, pOGG130, pOGG157 and pOGG024. The final *eYFP* module was amplified from pOPS0696 using primers oxp1842 and oxp1843 and cloned via HiFi reaction into pUC18T-mini-Tn7T-Gm digested with *Bam*HI and *Pst*I.

All plasmids were mobilized into *Rlv* OPS2038 by triparental mating, using the Tn*7* transposase-encoding plasmid pTNS3. Transposition events were selected on TY supplemented with gentamicin, and final strains were verified by PCR using primer pairs oxp2327/oxp3060 amplifying the left Tn*7* end and oxp1908/oxp3061 amplifying the right end of the Tn*7* integration.

### Recombinant protein expression and purification

Both *aatA* and *_Ec_aspA* (from strain DH5α) were PCR amplified from gDNA using primer pairs p405/p406 and p505/p506, respectively, and were inserted into pET101/D-TOPO (Invitrogen) using topoisomerase-based (TOPO) cloning. The resulting plasmids pRU1152 and pRU1741 were transformed into *E. coli* BL21 Star (DE3). Twenty-five millilitres of overnight culture were used to inoculate 500 ml liquid LB medium and grown to an OD_600_=0.5–0.8 when protein expression was induced by the addition of IPTG (final concentration of 1 mM). After 4–6 h at 37 °C, cells were harvested by centrifugation (10 min, 3000 ***g***, 4 °C). All the following steps were carried out at 4 °C: cells were disrupted in 1× His-Trap buffer by two passages through high-pressure homogenization in volumes of 5–10 ml at 69 MPa. Proteins were purified using an AKTA basic 10 (Amersham) and a 1 ml His-Trap column (Amersham). The column was prepared according to the manufacturer’s recommendations, except that imidazole was replaced with histidine, as imidazole was found to inactivate AatA. Crude extracts in 1× His-Trap buffer were centrifuged and filter sterilized and then loaded onto the His-Trap column. Proteins were eluted using a histidine gradient ranging from 0.5 to 100 mM in 0.5 M NaCl and 20 mM Tris-HCl at pH 7.9. The purity of AatA and AspC fractions was tested on SDS gels. Protein concentrations were determined using Bradford assays.

### Enzyme assays

For crude extract assays, wild-type A34 and the *aatA* mutant RU1640 were grown in TY medium to an OD_600_=0.5; cells were washed in 10 mM HEPES at pH 7.2, resuspended in 40 mM HEPES at pH 7.2, 2 mM DTT and 20 % glycerol and lysed in a cold French press. Lysed cells were spun down two to three times at 47 000 ***g*** for 45 min at 4 °C until no further pellet precipitated. The final clear supernatant was used as an enzyme extract.

All transamination assays were performed as linked assays, where the ketoacid product of the reaction acts as a substrate for a reduced nicotinamide adenine dinucleotide (NADH) consuming enzyme, and the reaction can be followed spectrophotometrically at OD_340_. Reactions took place in 1-ml cuvettes at 30 °C. All assays were performed in 100 mM phosphate buffer at pH 7.5 if not stated otherwise with the following additions depending on the assay: GOT assay [0.2 mM NADH, 0.05 mM pyridoxal 5'-phosphate (PLP), 1.8 units of l-glutamic dehydrogenase (Gdh) (Sigma G-2626), 100 mM NH_4_Cl and 1 mM oxaloacetate; 50 mM glutamate was added to start the reaction], AKT assay [0.2 mM NADH, 0.05 mM PLP, 5 units of malic dehydrogenase (Mdh) (Sigma M-2634) and 10 mM α-ketoglutarate; 50 mM aspartate was added to start the reaction], APT assay (0.2 mM NADH, 0.05 mM PLP, 5 units of Mdh and 75 mM pyruvate; 50 mM aspartate was added to start the reaction) and GPT assay [0.2 mM NADH, 0.05 mM PLP, 1.8 units of Gdh (Sigma G-2626), 100 mM NH_4_Cl and 75 mM pyruvate; 50 mM glutamate was added to start the reaction]. A total of 10 mM cyanide was added if crude extracts were assayed. Mdh was omitted in crude extract assays due to its natural presence.

### Structure modelling

*Rlv* AatA structure predictions were generated using ColabFold [31]. Structural alignments and analyses were carried out using PyMOL (Molecular Graphics System, Version 2.3.3 Schrödinger, LLC).

### Plant assays

Seed sterilization, plant growth and acetylene reduction assays were done as described in [32], except that plants were grown for 21 days.

## Results

### AatA is a bifunctional enzyme

**Fig. 2.**
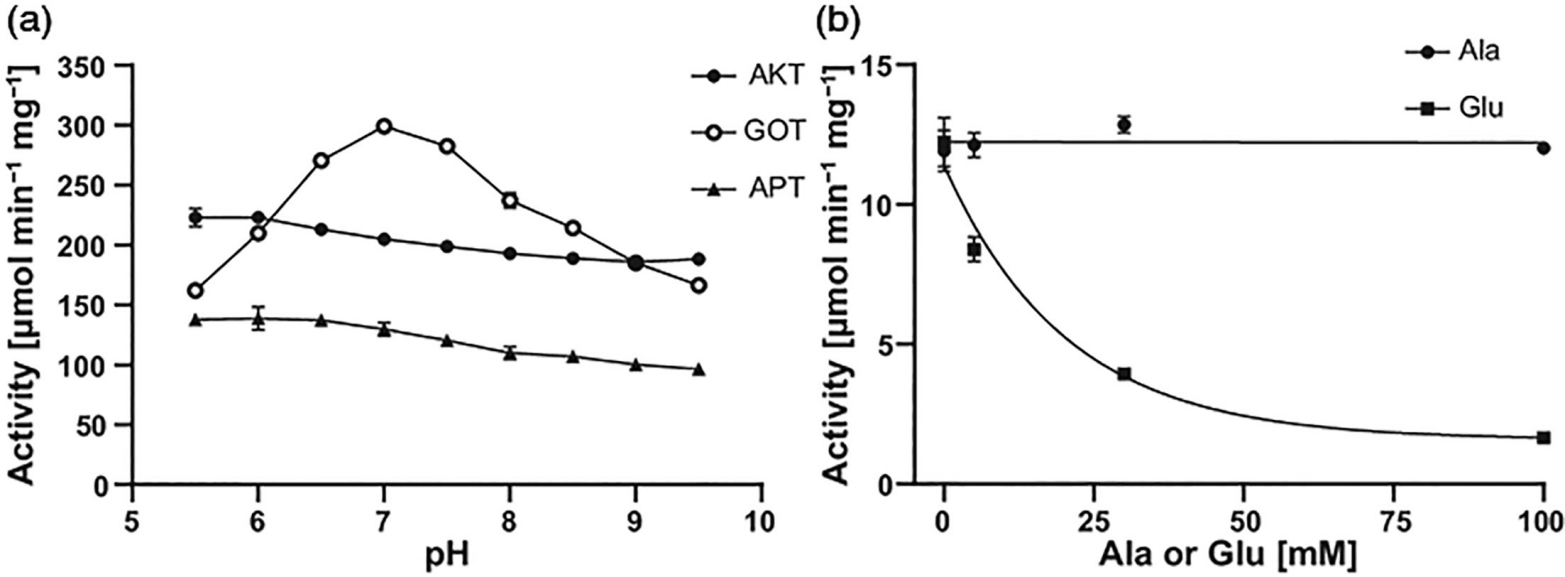
AatA activities. (**a**) Effect of pH on purified AatA of *R. leguminosarum* bv. *viciae* A34 was tested for AKT, GOT and APT activities. A total of 30 mM of MES (pH 5.5–6.5), HEPES (pH 7.0–8.0) or AMPSO (N-(1,1-Dimethyl-2-hydroxyethyl)-3-amino-2-hydroxypropanesulfonic acid) (pH 8.5–9.5) was used in the adjusted reaction mixtures. (**b**) Inhibition of APT activity by either alanine or glutamate was measured in low aspartate (2 mM) and pyruvate (5 mM) conditions. *n*=3 (**a**) or *n*=2 (**b**). GraphPad Prism 9.4.1 was used to determine one-phase decay nonlinear fit (*R*^2^=0.9903) for glutamate inhibition or robust standard deviation of the residuals (RSDR=0.4107) for alanine inhibition.

When crude extracts of wild-type *Rlv* A34 were assayed for GPT activity (transferring the amino group from glutamate onto pyruvate, forming α-ketoglutarate and alanine, Fig. 1), no significant activity was found (0.008±0.004 µmol min^−1^ mg^−1^; *n*=3), in accordance with the genome lacking genes encoding such enzymes. However, both AKT activity (transferring the amino group of aspartate onto α-ketoglutarate, forming glutamate and oxaloacetate) and APT activity (transferring the amino group of aspartate onto pyruvate, forming alanine and oxaloacetate) were found (Table 2). As AKT in *Rlv* is encoded by *aatA*, we tested crude extracts of an *aatA* mutant for the same enzyme activities and found that not only AKT activity but also APT activity was lost (Table 2). We thus reasoned that AatA might be a bifunctional enzyme, capable of transferring the amino group of aspartate onto both pyruvate and α-ketoglutarate. To further confirm these activities, we purified the recombinant A34 AatA and tested the isolated enzyme for GOT (reverse AKT activity), AKT and APT activity (Fig. 2a). AatA showed both AKT and GOT activities, as was expected from an aspartate aminotransferase. However, in addition, AatA indeed also showed considerable APT activity, at around 60 % of its AKT activity. Notably, GOT activity showed a considerable dependency on pH, with maximum activity at neutral pH, whereas both AKT and APT activities were barely affected by pH (Fig. 2a). Due to technical limitations, reverse APT activity (transferring the amino group of alanine onto oxaloacetate, forming aspartate and pyruvate) could not be measured, as the specific combination of alanine and oxaloacetate in the reaction mixture interfered with the NADH-consuming linking enzyme lactate dehydrogenase, which was intended to measure pyruvate release. We thus resorted to testing whether alanine (a product of the APT reaction) might inhibit the APT activity due to substrate-binding site competition on AatA. Increasing amounts of alanine were added to APT reaction mixtures with low amounts of the two APT substrates aspartate (2 mM) and pyruvate (5 mM). No inhibition of the APT activity was observable up to an addition of 100 mM alanine. In contrast, increasing the amounts of the known AatA substrate glutamate in the APT reaction mixtures caused considerable APT activity reduction (Fig. 2b). Thus, alanine does not appear to bind strongly to the active site of AatA, and we concluded that reverse APT activity is likely to be minimal and negligible in a physiological context.

**Table 2.** AKT and APT activities in *Rlv* wild-type (A34) and *aatA*-mutant crude extracts

Strain	AKT	APT
A34	0.451±0.157	0.194±0.056
*aatA*	0.020±0.005	0.013±0.002

A34, wild-type; AKT, aspartate–α-ketoglutarate transaminase activity; APT, aspartate–pyruvate transaminase activityA34: Wild type; AKT: aspartate α-ketoglutarate transaminase activity; APT: and aspartate pyruvate transaminase activity; aAll activities given in µmol min−1 mg−1. Number of replicates for each assay were *n*=3 (A34 AKT), *n*=5 (A34 APT and *aatA* AKT), and *n*=4 (*aatA* APT).

AatA was further characterized by measuring the Michaelis–Menten kinetics as reactions of pseudo-first orders for AKT (Fig. S1), GOT (Fig. S2) and APT (Fig. S3) reactions. *K*_m_, *V*_max_, *k*_cat_ and *k*_A_ values were determined for both substrates (donor amino acid and carboxylic acid) in each reaction (Table 3). For both AKT and GOT reactions, AatA showed a higher affinity for the carboxylic substrate than for the amino acids aspartate and glutamate, which only bind to AatA with *K*_m_ values in the millimolar range. Affinities for the C_4_ substrates aspartate and oxaloacetate were higher than for the C_5_ substrates glutamate and α-ketoglutarate, respectively. GOT activity was slightly higher than AKT activity. *V*_max_ and *k*_cat_ of the APT reaction were found to be lower than for the AKT reaction both at around 70 % of the latter, confirming the previously found activity in crude extracts. The affinity for aspartate was similar for both APT and AKT reactions; however, affinity for the C_3_ substrate pyruvate was found to be two orders of magnitude lower than for oxaloacetate or α-ketoglutarate. Both *k*_cat_ and *V*_max_ values were consistent for each reaction between the amino acid and carboxylic acid substrates. Our measured AKT and GOT kinetics of AatA are in the same range as values measured previously for other bacterial AatAs [33,36].

**Table 3.** AatA enzyme parameters

Reaction	Substrate	*K*_m_ (mM)	*V*_max_ (μmol s^−1^)	*k*_cat_ (s^−1^)	*k*_A_ (mM^−1^ s^−1^)
GOT	Glutamate	4.43±0.52	3.75±0.08	163.71±3.64	36.96±4.41
	Oxaloacetate	0.18±0.03	3.85±0.15	168.08±6.55	913.47±148.31
AKT	Aspartate	1.74±0.32	2.88±0.12	125.88±5.09	72.34±13.62
	α-Ketoglutarate	0.32±0.02	3.00±0.05	130.97±2.18	404.23±19.89
APT	Aspartate	1.52±0.09	1.81±0.02	79.31±0.73	52.18±3.13
	Pyruvate	17.0±2.00	2.27±0.10	98.96±4.37	5.82±0.73

Underlying data for this table can be found in supplementary fFigures. S1, S2, and S3.

Overall, we have shown that AatA of *Rlv* is a bifunctional enzyme, with the expected AKT and GOT activities but additional APT activity. Whilst *Rlv* lacks any GPT activity, AatA still can couple alanine biosynthesis to glutamate via the intermediate step of aspartate biosynthesis.

### AatA is highly similar to *E. coli* AspC but lacks one active-site arginine residue

**Fig. 3.**
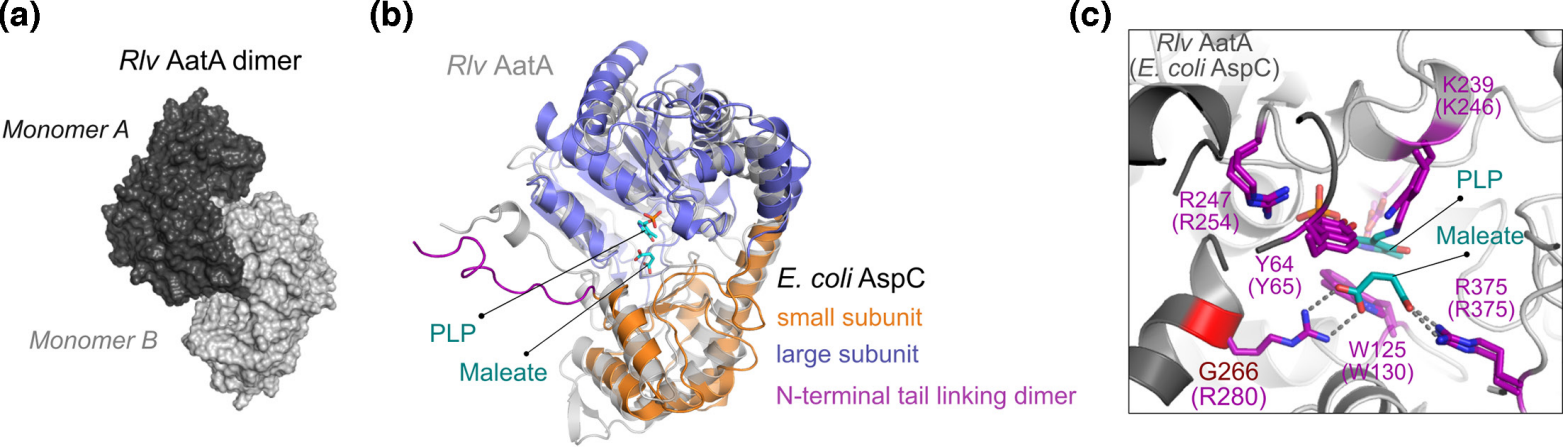
Comparison of structures of *R. leguminosarum* bv. *viciae* (*Rlv*) AatA and *E. coli* AspC. (**a**) *Rlv* AatA catalyses reactions by forming a dimer with two identical subunits composing the active site of the enzyme. Surface representation of an AlphaFold2 model is shown. (**b**) Overlay of the monomeric subunits of *Rlv* AatA with *E. coli* AspC (PDB ID: 8E9K) shows a high overall structural similarity (r.m.s.d=3.84 Å). Cartoon-stick models are shown with structural features highlighted on *E. coli* AspC. The small subunit represents a flexible region shifting the enzyme from an ‘open’ to a ‘closed’ conformation upon binding of the substrates (here: PLP and maleate, an AatA inhibitor) by the large subunit. The N-terminal tail links and stabilizes the dimeric form of AspC/AatA. (**c**) Enlarged view on the active site of *Rlv* AatA reveals the conservation of catalytically relevant residues apart from *E. coli* AspC R280 that coordinates the carboxyl group of dicarboxylate substrates including aspartate, which is replaced by G266 in *Rlv* AatA. Cartoon-stick model is shown of *Rlv* AatA with an overlay of catalytic residues of *E. coli* AspC (residue numbering in brackets) in stick representation coloured in magenta.

We have modelled AatA in its expected homo-dimeric structure using ColabFold (Fig. 3a). When overlayed with the solved crystal structure of the *E. coli* aspartate aminotransferase AspC (PDB ID: 8E9K) [37], we found that both enzymes are highly similar (root mean square deviation of atomic positions (r.m.s.d)=3.84 Å, Fig. 3b), with all active-site amino acid residues [38] conserved, except for arginine 280 of AspC. At the corresponding position, *Rlv* AatA possesses a glycine residue rather than an arginine (Fig. 3c).

### Deletion of AatA results in attenuated growth but not Asp auxotrophy

**Fig. 4.**
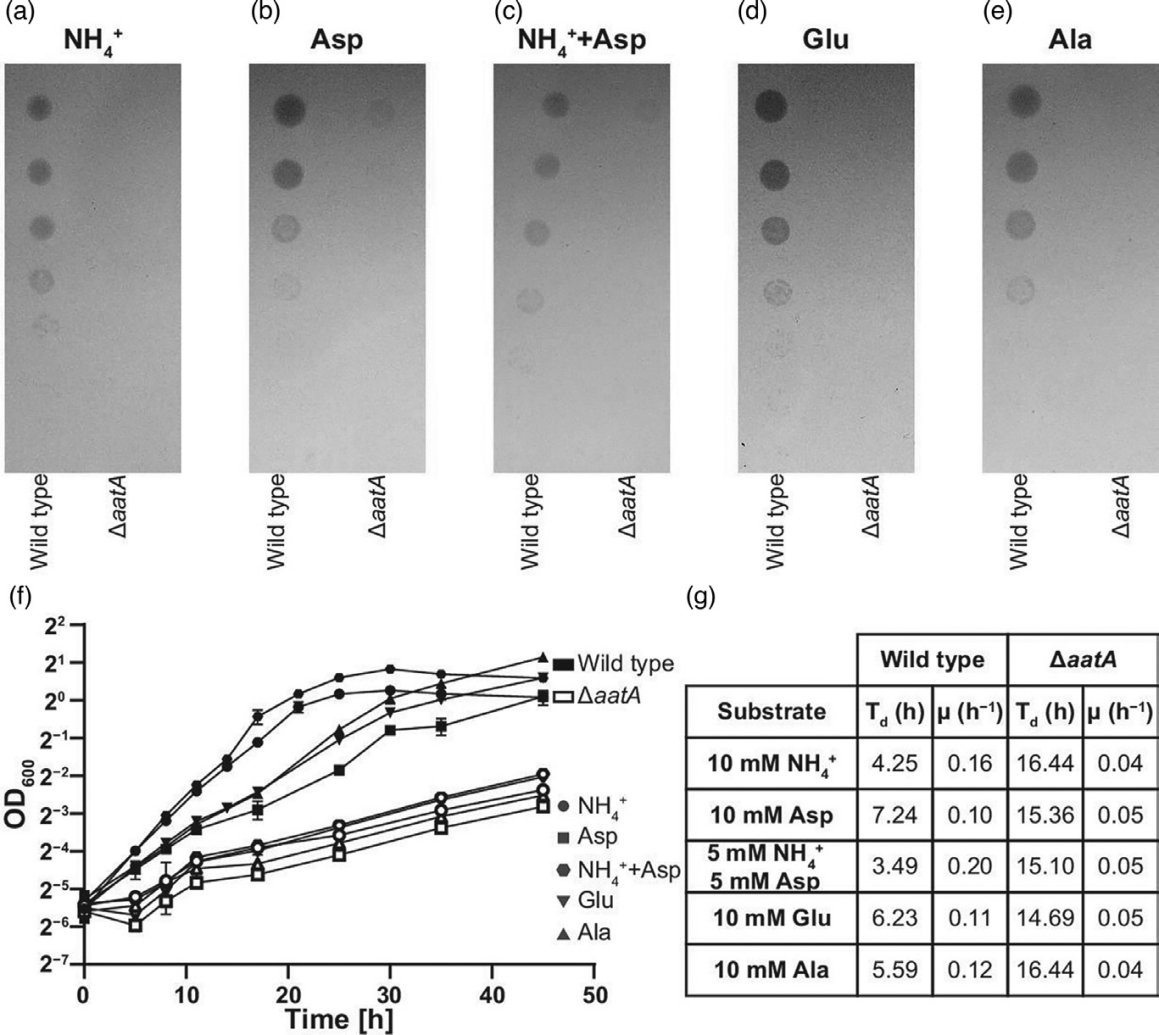
Effects of *aatA* deletion on free-living *R. leguminosarum* bv. *viciae* (*Rlv*) A34. Four microlitres of cell suspensions of *Rlv* wild-type and Δ*aatA* mutant were spotted in serial tenfold dilutions starting from OD_600_=0.1 onto UMS minimal medium plates supplemented with 20 mM succinate as a carbon source, and growth was tested depending on the provided nitrogen sources, either (**a**) 10 mM ammonium chloride, (**b**) 10 mM aspartate, (**c**) 5 mM ammonium chloride and 5 mM aspartate, (**d**) 10 mM glutamate or (**e**) 10 mM alanine as nitrogen source(s). (**f**) Wild-type and Δ*aatA* mutant were inoculated into liquid UMS minimal medium, supplemented with the above concentrations of carbon and nitrogen sources at an initial OD_600_=0.02. Growth was measured based on optical density changes at regular intervals and is presented as growth curves on a semi-log plot using log_2_. (**g**) Doubling times (*T*_d_) and specific growth rates (*µ*) during exponential growth were calculated for both strains under each condition. Each datapoint in (f) is derived from three biological replicates with sd shown.

A Δ*aatA* deletion mutant in *Rlv* A34 was found to exhibit severely impaired growth on minimal medium with either succinate or glucose as a carbon source (Figs 4a, f, g and S4A). AatA catalyses aspartate biosynthesis from glutamate (GOT activity), and consequently, it was suspected that a Δ*aatA* mutant could be an aspartate auxotrophic strain or, at least, suffer from inadequate aspartate biosynthesis. However, supplementing minimal growth medium with aspartate did not complement the mutant phenotype (Fig. 4b, f, g). As AatA is also involved in aspartate catabolism (via biosynthesis of glutamate and alanine from aspartate), growth on aspartate as a sole nitrogen source could have resulted in inadequate glutamate biosynthesis, thus masking the effect of aspartate supplementation. However, growth on minimal medium could neither be restored by the addition of aspartate and ammonium (Fig. 4c, g, f), which could be assimilated into glutamine via GS-GOGAT. Supplementing minimal medium with either glutamate or alanine did not complement the mutant phenotype either (Fig. 4d–g), as glutamate and alanine can be synthesized by other means than AatA. Importantly, a Δ*aatA* mutant grew better on complex TY medium than on minimal medium, yet it still displayed a substantially reduced fitness compared to the wild-type (Fig. S4B).

Hence, it appears that the fitness cost associated with the *aatA* deletion cannot be attributed to any apparent auxotrophy but might be rather due to internal metabolite pool size or flux control.

### Δ*aatA* can be complemented by heterologous aspartase expression

**Fig. 5.**
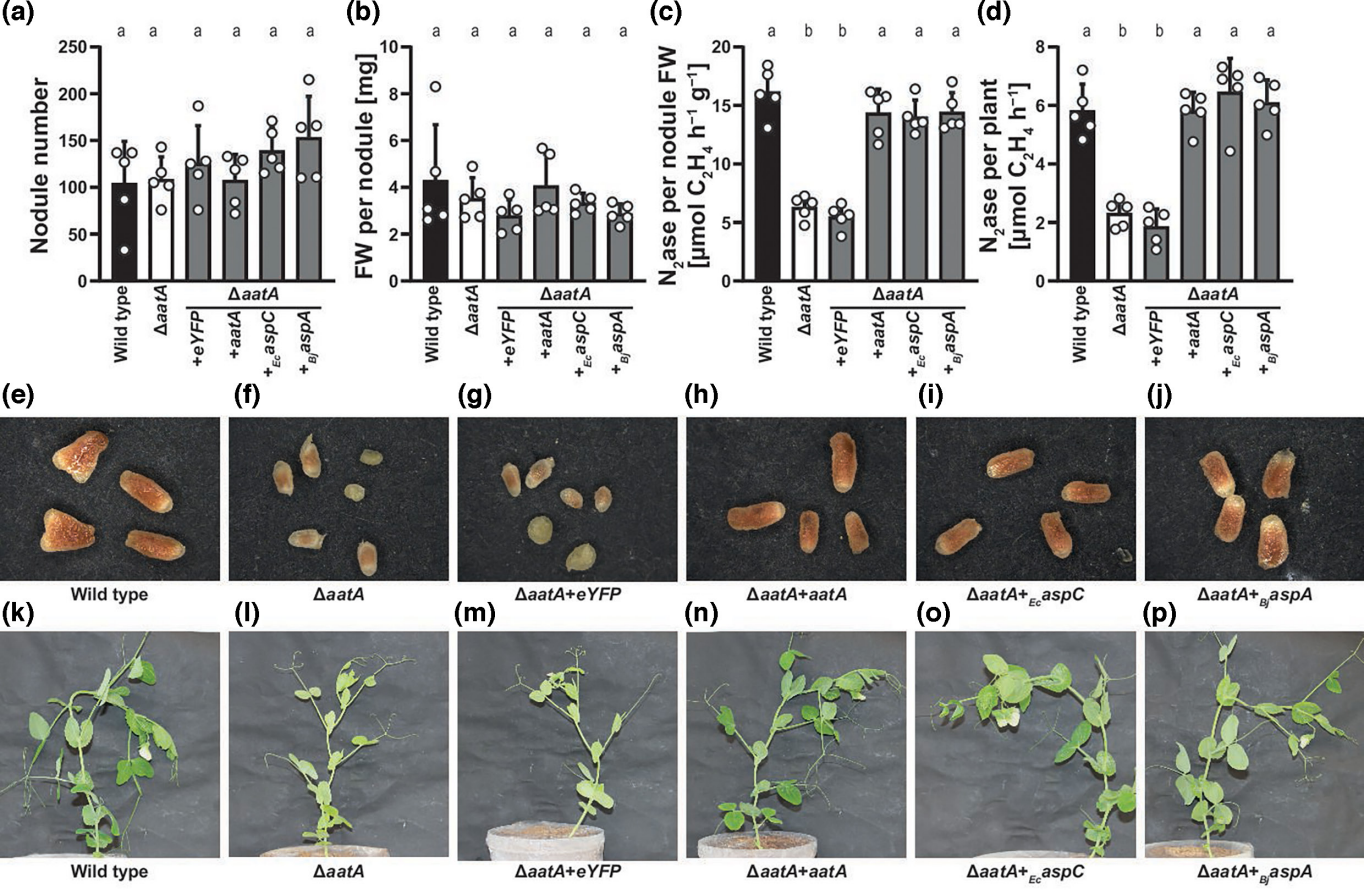
Complementation of the Δ*aatA*-mutant phenotype during symbiosis with pea plants. *Pisum sativum* cv. Avola seedlings were inoculated with wild-type, Δ*aatA* mutant or Δ*aatA* mutant expressing e*YFP*, *aatA*, *E. coli aspC* or *B. japonicum aspA*. Plants were assayed for (**a**) nodule number, (**b**) fresh weight (FW) per nodule, (**c**) nitrogenase activity per nodule fresh weight and (**d**) nitrogenase activity per plant. (**e–j**) Nodules cross sections of crown nodules of plants inoculated with the above-mentioned strains and (**k–p**) exemplary plants. Each strain was tested on *n*=5 plants. Different letters in (a)–(d) denote statistical differences (*α*≤0.05) as determined by Brown–Forsythe and Welch ANOVA tests, using Dunnett T3 multiple comparison correction. All column means were compared with each other mean.

A *aatA* mutant has previously been shown to be drastically affected in symbiosis with pea plants [18], but an explanation for this phenotype so far has not been found. To better understand which AatA activity is involved in symbiosis, we decided to suppress the *aatA* deletion-mutant phenotype by expressing *aspC* of *E. coli*. AspC is the homologue of *Rlv* A34 AatA but was found to lack APT activity (Table S2). Additionally, we also expressed *aspA* from *B. japonicum*, encoding an aspartate-catabolizing aspartase, which deaminates aspartate to fumarate and ammonium [39]. When we inoculated the resulting strains onto pea plants, we found that both *E. coli aspC* and *B. japonicum aspA* restored a wild-type-like phenotype in a Δ*aatA*-mutant background. Plants inoculated with the complemented strains showed restored nitrogenase activity and had deep-red nodules, and plants developed dark green leaves as opposed to the chlorotic phenotype of Δ*aatA*-mutant inoculated plants (Fig. 5), which is indicative of a nitrogen-starved physiology.

## Discussion

We have shown that in *Rlv*, AatA is a bifunctional enzyme, which not only catalyses the reversible transamination of aspartate to α-ketoglutarate (AKT and GOT reactions) but also has an extended substrate specificity. As well as the C_4_ and C_5_ dicarboxylates oxaloacetate and α-ketoglutarate, it could also accept the C_3_ monocarboxylate pyruvate as an acceptor for the aspartate amino group, forming alanine (APT reaction). Whilst the *K*_m_ of AatA for pyruvate was much lower than for oxaloacetate and α-ketoglutarate, the APT reaction still ran at around 60–70 % of the AKT reaction rate (Table 3 and Table S2). However, this secondary reaction was not the reason for the symbiotic phenotype of a Δ*aatA* mutant, as it could be complemented by the expression of the *E. coli* aspartate aminotransferase gene *aspC*. AspC does not show any APT reactivity (Table S2), indicating that the reversible AKT/GOT reaction was sufficient to restore a wild-type-like phenotype of a Δ*aatA* mutant in symbiosis with pea plants. Additionally, the lack of AatA in symbiosis could also be overcome by the expression of the *B. japonicum* aspartase gene *aspA*. Aspartases (aspartate ammonia lyases) catalyse the reversible deamination of aspartate to fumarate and ammonium. However, the physiological role of aspartases *in vivo* is clearly of catabolic nature [40,43], and they play no role in ammonia assimilation [44]. In addition, our free-living experiments indicated that a Δ*aatA* mutant is not an aspartate auxotrophic strain (Fig. 4), as the addition of aspartate to Δ*aatA*-mutant cultures did not complement the mutant’s phenotype. Moreover, a *Sinorhizobium meliloti* Δ*aatA* mutant was likewise affected in nitrogen fixation with its cognate host plant alfalfa (*Medicago sativa*) and could not grow on aspartate, indicating that AatA is needed in bacteroids due to its aspartate catabolic activity [22]. Together, this suggests that aspartate metabolism other than biosynthesis is essential for nitrogen fixation in bacteroids. It remains speculative why aspartate degradation is needed in bacteroids; aspartate could either build up to toxic levels if not catabolized efficiently, or aspartate could act as a regulatory molecule affecting other physiological processes. Likewise, the source of aspartate in bacteroids remains unknown. Aspartate only makes up around 0.6 % of all amino acids in bacteroids, but biosynthesis rates are high compared to most other amino acids with the exceptions of glutamate, γ-aminobutyric acid (GABA) and alanine [12]. It is possible that aspartate could be a catabolic product of asparagine, which comprises 73.2 and 45.6 % of amino acids in pea nodules and pea bacteroids, respectively [12]. Similar levels of asparagine can be expected in *S. meliloti* cells, as both host plants, pea and alfalfa, are amino acid-exporting plants using asparagine as a means to transport fixed nitrogen from the root to the shoot [45], explaining the sheer abundance found in nodules. However, other plants, including soybean, export ureides [46] rather than asparagine. Nonetheless, *aspA*, which we used to complement our Δ*aatA* mutant, was likewise found to be essential for nitrogen fixation in the soybean symbiont *B. japonicum* [39], hinting towards a conserved need for aspartate degradation in different rhizobia–legume symbioses. In such a scenario, AatA could metabolize aspartate to either glutamate or alanine. The latter is also a major N-export product (besides ammonium) produced by bacteroids and provided to the plant, especially under the low oxygen concentrations found in nodules [11,15, 16, 47]. Thus, the extended substrate specificity of AatA compared to *E. coli* AspC could provide an advantage to bacteroids. Notably, we have seen differences in the severity of nitrogen fixation reduction of *aatA* mutants ranging from drastic reduction (Fig. 5) to complete absence [18]. These differences are likely due to the status of the host plant, caused by different growth conditions, such as temperature, light intensity or plant age, as experiments were performed at different places. This indicates that the need for aspartate degradation in bacteroids is a direct result of the integrated metabolism of the symbiont and the host plant and that the physiological status of the latter has a pronounced effect on the amino acid metabolism of the symbiont. Moreover, the unique APT activity of AatA also enables *Rlv* to directly couple the pools of glutamate, aspartate and alanine via transamination reactions with a single enzyme without the need for GPT enzymes, such as AlaA or AlaC in *E. coli* [7]. The structure of aspartate aminotransferases has been extensively studied [48,49], in both eukaryotic [50] and mitochondrial [51] versions of animals, as well as in *E. coli* [52]. All aspartate aminotransferases are highly conserved with several residues recognized as participating in and essential for the catalysed reactions [38]. Aspartate aminostransferases are pyridoxal phosphate-dependent enzymes, which work functionally as dimers (Fig. 3). Particularly important are residues R375 and R280 (*E. coli* AspC polypeptide, in older literature usually referred to as R386 and R292, respectively), which are part of the active site and responsible for binding the two carboxyl groups of the substrate dicarboxylates oxaloacetate and α-ketoglutarate [53]. *E. coli* AspC R375 is responsible for binding the α-carboxyl group, whereas R280 binds the side-chain carboxyl group. Mutational analyses of R280 in *E. coli* AspC have shown that this residue is responsible for the specificity towards dicarboxylates, as when the residue is replaced with other amino acids, the specificity of the enzyme changes, depending on the amino acid substitution [54,56]. When a neutral amino acid is used to replace R280, its specificity changes from aspartate and glutamate to transamination reactions favouring neutral amino acids [57]. Strikingly, at the corresponding position of *Rlv* AatA, a glycine (G266) is found (Fig. 3c). Thus, AatA’s specificity for the monocarboxylate pyruvate can be partially explained by the presence of this residue where other aspartate aminotransferases possess an arginine residue for complexation of the second carboxyl residue in cognate dicarboxylate substrates. However, mutations of R280 abolished the activity of *E. coli* AspC for either C_4_ or C_5_ dicarboxylates [57]. How exactly *Rlv* AatA maintains its specificity as a genuine aspartate aminotransferase (Table 3), despite the lack of arginine residue, remains unclear. Nonetheless, the extended substrate specificity of *Rlv* AatA may have significant physiological impacts on the lifestyle of *Rhizobium* and also presents the first description of such an enzyme, capable of balancing the specificity for the negatively charged amino acids aspartate and glutamate whilst showing additional reactivity towards biosynthesis of the neutral amino acid alanine.

Aspartate and glutamate are central to the amino acid metabolism. The extended substrate specificity of AatA may thus have significant implications on the amino acid metabolism, such as pool sizes and fluxes of *Rlv*, as implied by the associated free-living phenotypes (Figs. 4 and S4). Moreover, the role of AatA for nitrogen fixation in bacteroids makes this enzyme a clear target for future experiments. As the phenotype of *aatA* mutants seems to differ based on the physiological status of the plant, AatA is likely involved in balancing the endogenous aspartate, and by extension, wider amino acid pools in response to the host physiological status. As amino acids are not only secreted by the bacteroids (including aspartate and alanine) [16,19,21, 47] but also received from the plant [23,24], AatA is likely involved in integrating the symbiont into the host metabolism. Metabolomic analysis of the mutant in free-living conditions but also from bacteroids may elucidate the exact role of AatA on the wider metabolic landscape under these conditions.

## supplementary material

10.1099/mic.0.001471 Uncited Supplementary Material 1.

## Abbreviations

AatA: aspartate aminotransferase
AKT: aspartate-α-ketoglutarate transamination
AldA: alanine dehydrogenase
AMPSO: N-(1,1-Dimethyl-2-hydroxyethyl)-3-amino-2-hydroxypropanesulfonic acid
APT: aspartate–pyruvate transamination
GABA: γ-aminobutyric acid
GOGAT: glutamine oxoglutarate transaminase
GOT: glutamate–oxaloacetate transamination
GPT: glutamate–pyruvate transamination
GS: glutamine synthase
LB: lysogeny broth
NADH: nicotinamide adenine dinucleotide
PLP: pyridoxal 5'-phosphate
*Rlv*: *Rhizobium leguminosarum* bv*. viciae*
r.m.s.d: root mean square deviation of atomic positions
RSDR: robust standard deviation of the residuals
TOPO: topoisomerase-based
TY: tryptone - yeast extract
UMS: universal minimal salts
