# Supplementary material for: Aspartate aminotransferase of Rhizobium leguminosarum has extended substrate specificity and metabolizes aspartate to enable N2 fixation in pea nodules
Source: Microbiology (Reading). 2024 Jul 29;170(7):001471. doi: 10.1099/mic.0.001471 (PMC11286295; doi:10.1099/mic.0.001471)
Supplement: Uncited Supplementary Material 1. [file mic-170-01471-s001.pdf]

Supplementary Material for

Aspartate aminotransferase of *Rhizobium leguminosarum* has extended substrate specificity and metabolises aspartate to enable N_2-fixation in pea nodules

Ledermann R.[1], Bourdès A.[2,3], Schuller M.[4], Jorrin B.[1], Ahel I.[4], and Poole P. S.[1,2,3]

[1] Department of Biology, University of Oxford, OX1 3RB, Oxford, United Kingdom

[2] John Innes Centre, NR4 7UH, Norwich, United Kingdom

[3] School of Animal and Microbial Sciences, University of Reading, RG6 6AJ, Reading, United Kingdom

[4] Sir William Dunn School of Pathology, University of Oxford, OX1 3RE, Oxford, United Kingdom

Supplementary Tables

Table S1: Primers used in this study

Name	Sequence 5'-3'
oxp1908	CGAGAAAATTATTCCTCAGCCGG
oxp1842	CATGCATGAGCTCACTAGTGCTCTAGGGCGGCGG
oxp1843	CTTCTCGAGGAATTCCTGCAGCTGGATTCTCACCAATAAAAAACG
oxp2327	GATGATCTTCTCGCTGCCGA
oxp3060	CACAGCATAACTGGACTGATTTC
oxp3061	ATTAGCTTACGACGCTACACCC
oxp3528	TTCGCGAGGTACCGGGCCCATTACAGCACTGCCACAATC
oxp3529	CAACCATTAGGACCGTTATCATGTTTGAGAACATTACCGC
oxp3534	GATAACGGTCCTAATGGTTGAAACGGGCAG
oxp3535	CATGCATGAGCTCACTAGTGTGGCATACCCTCAATAAC
oxp3536	TTCGCGAGGTACCGGGCCCATTACTTGCAGGCGCCGCA
oxp3537	CAACCATTAGGACCGTTATCATGGCTTTCCTTGCCGATGC
oxp3538	CGGTGGCGGCCGCTCTAGAAGGAATTGTCCGGCAGTGG
oxp3539	CGCCACTAGTAAGGAAAGCCATGATAACGG
oxp3540	GGCTTTCCTTACTAGTGGCGCCTGCAAGTAAGCG
oxp3541	CGAATTCCTGCAGCCCGGGGGCACGCCGGCATTGAAATAG
oxp3638	AGATCTCCCGTGTCCTGGC
oxp3639	TGCTCGTAGATCTCGCGGG
oxp3640	GCCTTCCGATTCCAGCAGC
oxp3641	ATCAACCGCGGCGAAACG
oxp3753	CAACCATTAGGACCGTTATCATGAGCCGTACGGAGCAG
oxp3754	TTCGCGAGGTACCGGGCCCATCACGCAATCAGGTCCGG
p405	CACCATGGCTTTCCTTGCCGATGCTC
p406	CTTGCAGGCGCCGCAGAAGCG
p505	CACCATGTTTGAGAACATTACCGCC
p506	CAGCACTGCCACAATCGCTTC

Table S2: Comparison between *Rlv* A34 AatA and *E. coli* AspC

Enzyme	AKT	APT	APT in % of AKT
AatA	183±6.0	110±1.8	61.1%
AspC	238±12	2.5±0.9	1.1%

n=3, all activities given in µmol min^{-1} mg^{-1}

Supplementary Figures

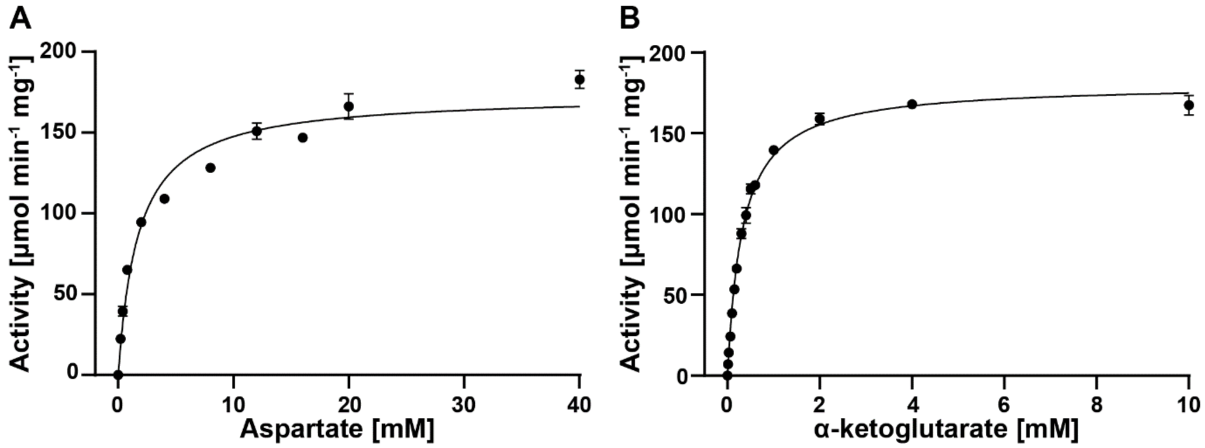

Fig. S1: Michaelis-Menten kinetics of AatA aspartate – α-ketoglutarate transamination reaction (AKT), using either varying concentrations of (A) aspartate or (B) α-ketoglutarate to generate a reaction of pseudo-first order. n=3 with standard deviation shown for each datapoint, nonlinear Michaelis-Menten kinetics were fitted (R^2=0.9753 and R^2=0.9965 for A and B, respectively) using GraphPad Prism 9.4.1.

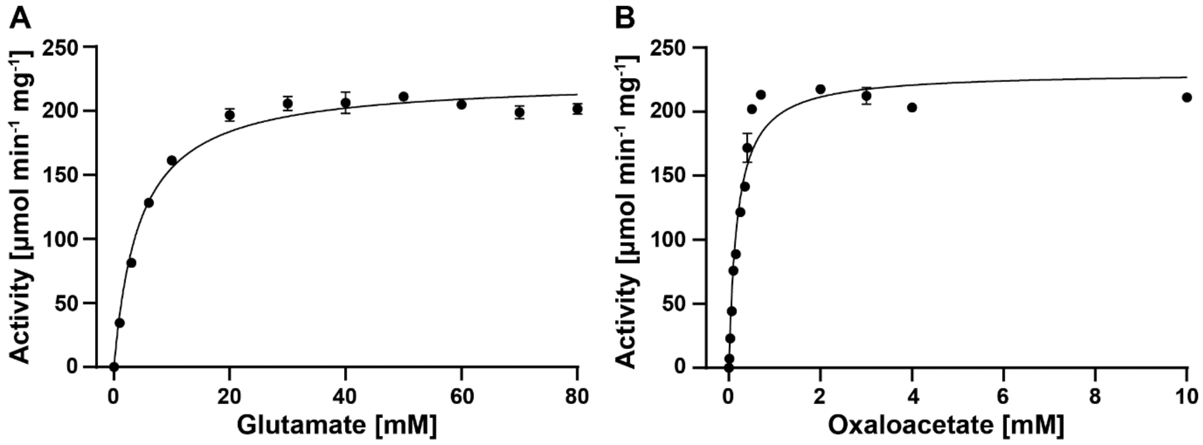

Fig. S2: Michaelis-Menten kinetics of AatA glutamate – oxaloacetate transamination reaction (GOT), using either varying concentrations of (A) glutamate or (B) oxaloacetate to generate a reaction of pseudo-first order. n=3 with standard deviation shown for each datapoint, nonlinear Michaelis-Menten kinetics were fitted (R^2=0.9875 and R^2=0.9628 for A and B, respectively) using GraphPad Prism 9.4.1.

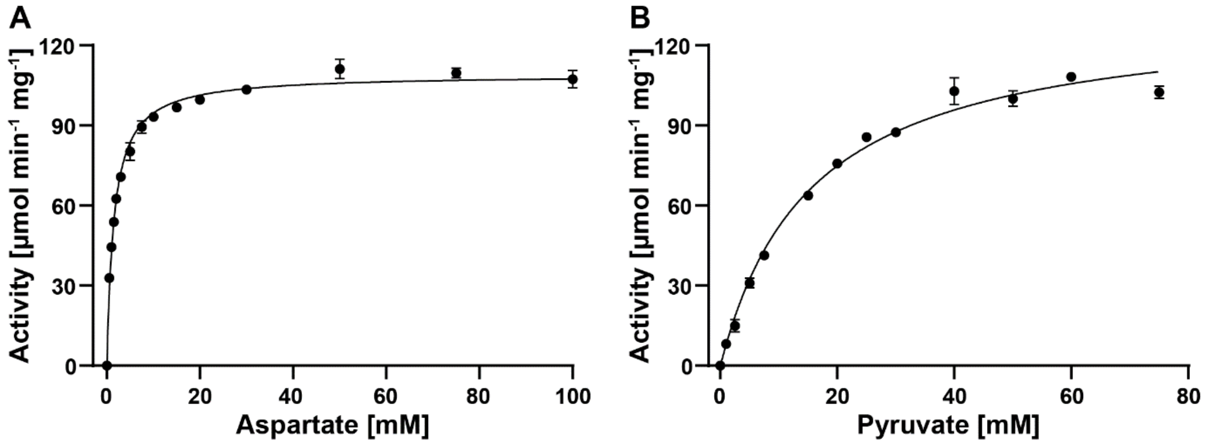

Fig. S3: Michaelis-Menten kinetics of AatA aspartate – pyruvate transamination reaction (APT), using either varying concentrations of (A) aspartate or (B) pyruvate to generate a reaction of pseudo-first order. n=3 with standard deviation shown for each datapoint, nonlinear Michaelis-Menten kinetics were fitted (R^2=0.9935 and R^2=0.9918 for A and B, respectively) using GraphPad Prism 9.4.1.

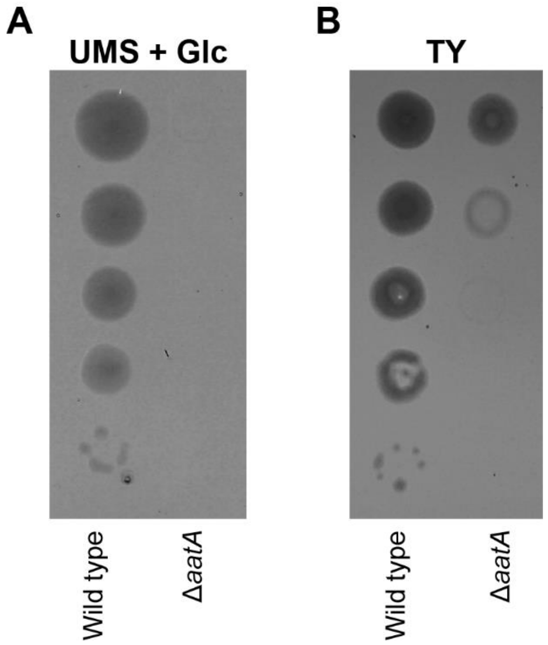

Fig. S4: Effects of *aatA* deletion on growth of *Rhizobium leguminosarum* bv. *viciae* (*Rlv*) A34 on (A) minimal medium supplemented with glucose and (B) complex TY medium. 4 µl of cell suspensions of *Rlv* wild type and Δ*aatA* mutant were spotted in serial 10-fold dilutions starting from OD_{600}=0.01 onto UMS minimal medium plates supplemented with 10 mM glucose as a carbon source or complex TY medium. For detailed growth characteristics on minimal medium with succinate as a carbon source, *cf.* Figure 4.
